# *Babesia* and *Bartonella* Species DNA in Blood and Enrichment Blood Cultures from People with Chronic Fatigue and Concurrent Neurological Symptoms

**DOI:** 10.3390/pathogens15010002

**Published:** 2025-12-19

**Authors:** Edward B. Breitschwerdt, Ricardo G. Maggi, Janice C. Bush, Emily Kingston

**Affiliations:** Intracellular Pathogens Research Laboratory, Comparative Medicine Institute, College of Veterinary Medicine, North Carolina State University, Raleigh, NC 27607, USA; rgmaggi@ncsu.edu (R.G.M.); jcbush@ncsu.edu (J.C.B.); emkingst@ncsu.edu (E.K.)

**Keywords:** *Bartonella*, *Babesia*, flea, tick, vector, infection, PCR, enrichment culture

## Abstract

Myalgic encephalomyelitis/chronic fatigue syndrome (ME/CFS) is a medical condition characterized by extreme fatigue lasting at least 6 months. Based upon case reports, patients infected with *Babesia* or *Bartonella* spp. have reported a history of chronic fatigue and concurrent neurological symptoms. In this study, 50 study participants reporting fatigue lasting from six months to 19 years and one or more neurological symptoms were selected. PCR assays were used to amplify *Babesia* and *Bartonella* spp. DNA from blood and enrichment blood cultures. Using targeted qPCR amplification and DNA sequencing, infection with *Babesia* spp., *Bartonella* spp. or both genera was confirmed in 10, 11, and 2 individuals, respectively. Of 50 participants, 12 (24%, 95% CI: 12–36%) were infected with a *Babesia* species, while *Bartonella* species infection was documented in 13/50 individuals (26%, 95% CI: 13.8–38.2%). This study provides documentation supporting a potential role for *Babesia* and *Bartonella* infection in patients with presentations consistent with ME/CFS. Prospective case–control studies, using highly sensitive direct pathogen detection techniques, are needed to determine whether or the extent to which infection with members of these two genera contributes to or causes ME/CFS.

## 1. Introduction

In recent years, bloodborne *Babesia* and *Bartonella* species infections or coinfections have been identified in patients with chronic, and often nonspecific illnesses [1,2,3]. Historically, the predominant clinical emphases for both genera have focused primarily on acute illness presentations, such as hemolytic anemia and/or thrombocytopenia for *Babesia* species and acute febrile illness (i.e., Oroya fever, Trench fever, Cat Scratch Fever) for *Bartonella* species. Documentation of pathogen DNA in the blood or tissues of patients with chronic illness, who lack the typical findings associated with acute illness, continues to challenge and change the collective medical understanding of disease dynamics, pathogenesis, and epidemiology for babesiosis, bartonellosis and co-infections with both genera. In addition to the diagnostic sensitivity limitations associated with *Babesia* and *Bartonella* spp. molecular testing, the lack of therapeutic guidelines for chronically infected patients can create diagnostic and treatment dilemmas for physicians.

Importantly, the use of more sensitive molecular diagnostic testing modalities has confirmed infection with both genera in transplant recipients and healthy blood donors [4,5,6,7], further illustrating the potential for both genera to induce a longstanding intravascular infection, as well as the need for expanded blood donor screening. In the context of blood transfusion considerations, there are no current *Bartonella* screening recommendations for asymptomatic human blood donors and *Babesia* screening occurs on a limited regional basis [4,6].

Ongoing developmental and technical improvements have resulted in more sensitive molecular diagnostic techniques, as confirmatory methods to assess “active” infection with *Babesia* and *Bartonella* species. Documentation of pathogen DNA in patient specimens (blood, cerebrospinal fluid, joint fluid, and pathological effusions) is providing increased clarity to the healthcare community as to the contribution of these organisms to chronic, nonspecific symptoms among individual patients. Using a combination of microbiological culture and molecular DNA amplification approaches, infection with *Babesia*, *Bartonella* and coinfections with both genera have been confirmed in people suffering chronic and often nonspecific symptoms that include debilitating fatigue and neurological symptoms [1,2,3,8,9]. Due to their ability to induce chronic subclinical infections, accompanied by low and potentially intermittent parasitemia or bacteremia, molecular confirmation of infection with these protozoa and bacteria remains diagnostically challenging. In addition, as compared to single infections, co-infections with *Babesia* and *Bartonella* spp. influence antimicrobial drug selection and treatment planning for patients.

Myalgic encephalomyelitis/chronic fatigue syndrome (ME/CFS) is a medical condition characterized by extreme fatigue lasting at least 6 months. According to the World Health Organization, ME/CFS impacts approximately 17 million people worldwide [10]. The syndrome often includes post-exertional malaise with worsening symptoms after minimal exercise, as well as a wide range of other symptoms, most often including cognitive impairment and orthostatic intolerance. Currently, there is no international consensus for diagnostic criteria for ME/CFS. In the context of infectious causation, research to date has focused primarily on viruses; however, no consistent evidence has supported a viral etiology [11]. The purpose of this study was to determine the frequency of *Babesia* and *Bartonella* spp. DNA detection in a cohort of research participants with a history of fatigue lasting at least six months.

## 2. Patients and Methods

### Study Population

Participants were selected from a cohort of 173 individuals who were previously tested for *Bartonella* spp. infection as a component of an Institutional Review Board (IRB) approved study entitled: Detection of *Bartonella* Species in the Blood of People with Extensive Animal Contact (North Carolina State University Institutional Review Board, IRB# 1960). Due to chronic illnesses of varying severity and duration, all participants or their physician had contacted the corresponding author requesting study entry. Individuals were included if they met two criteria: (1) based upon study questionnaire responses, they had experienced fatigue or chronic fatigue for a duration of at least six months and (2) they had reported (questionnaire checklist) one or more neurological symptoms, specifically including the following: difficulty remembering, disorientation, irritability, rage, aggression, difficulty sleeping, seizures, tremors, headache, mental confusion, hallucinations, and anxiety/panic attacks. Although the duration of illness varied among individuals, as did prior diagnostic evaluations and previous treatments, these factors were not criteria for study inclusion or exclusion. Questionnaires previously completed by study participants between February 2020 and March 2024 underwent a blinded sequential review to select individuals who satisfied entry criteria. The standardized questionnaire included age, gender, animal and arthropod exposure, outdoor activity, travel, clinical symptoms, duration of illness, co-morbid conditions and evaluation by specialist physicians.

Participants provided three blood specimens (triple blood draw) collected within a 7-day period, which were then cultured for 21 days, with sampling for DNA extraction at 7, 14, and 21 days. Stored blood and enrichment blood culture DNA from individuals satisfying the inclusion criteria were tested for DNA evidence of *Babesia* spp. infection. Molecular testing was performed using DNA extracted from blood and enrichment blood cultures. Of the 173 study participants tested between February 2020 and March 2024, 50 individuals met the inclusion criteria. The researcher (E. Kingston) reviewing the questionnaires for case selection based upon entry criteria and the individuals (R. Maggi and E. Kingston) performing the molecular testing were blinded to prior *Bartonella* or *Babesia* test results.

**Figure 1:**
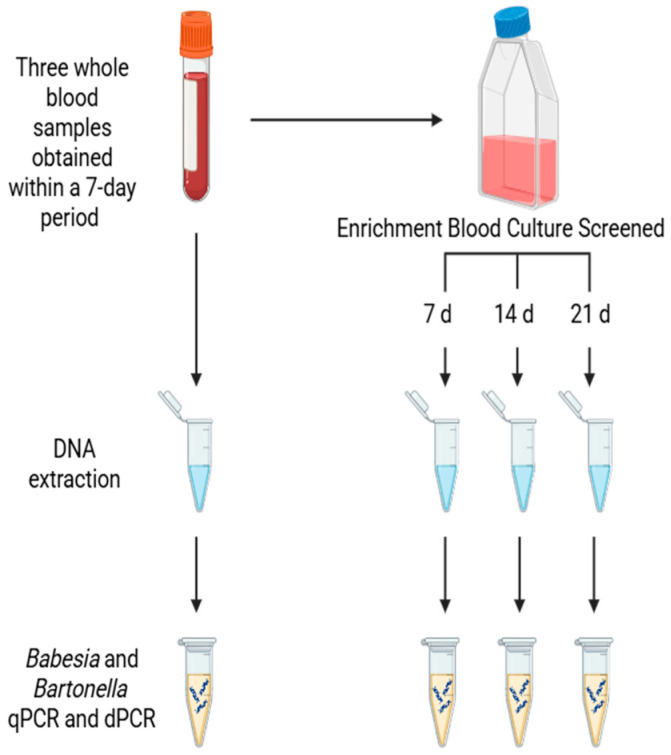
Sample processing for *Babesia* and *Bartonella* species detection in blood and enrichment blood cultures. Study participants were subjected to three blood draws, typically on a Monday, Wednesday and Friday, within a one-week period. Blood from each draw was both directly sampled for DNA extraction and placed into enrichment culture, which was then subject to DNA extraction for *Babesia* and *Bartonella* qPCR and dPCR amplification after 7, 14, and 21 days in incubation at 35 °C with 5% CO_2_.

The enrichment blood culture and molecular testing methods for *Bartonella* spp. detection have been described previously. The methods used in this study for *Babesia* and *Bartonella* spp. DNA amplification and DNA sequencing were recently published [3,12] with minor modifications in primer/probe design as listed in Table 1. Briefly, DNA extracted from blood and enrichment blood cultures (sampled at 7, 14, and 21 days) were screened for *Babesia* species amplification using quantitative real-time PCR (qPCR) targeting the 18SrRNA-5.8SrRNA intergenic spacer (ITS) region (Figure 1).

Real-time qPCR amplification of the *Babesia* ITS1 region was performed in a 25 μL reaction composed (per reaction) of 7.5 μL of molecular-grade water (QIAGEN, Germantown, MD, USA), 12.5 μL of 2X SsoAdvanced SYBR green master mix (Bio Rad, Hercules, CA, USA), 0.2 μL of 100 μM each oligonucleotide primers, and 5 μL of extracted DNA (used as template). Molecular-grade water and DNA extracted from naïve human blood samples were used as PCR negative controls. Pre-characterized DNA amplified from blood samples extracted from clinical cases of dogs infected with *Babesia gibsoni* was used as a positive control template. This species was selected not just for assessing PCR performance, but also to assess potential contamination/carry-over of *Babesia* DNA among patient samples during DNA amplification. Amplification was performed in a Bio-Rad CFX 96-well Opus PCR system machine (Hercules, CA, USA) under the following conditions: 95 °C for 3 min, followed by 40 cycles of denaturing at 94 °C for 15 s, annealing at 68 °C for 15 s, and extension at 72 °C for 15 s. An additional gradient cycle from 65 °C to 95 °C was performed for melting curve analysis. Quantification cycle (Cq) values and melting temperature analysis were performed by reading fluorescent signals using the SYBR/FAM, HEX, Texas-Red/TAMRA, and Cy5 fluorescent dye channels. To avoid PCR amplicon contamination during sample processing, a one-way workflow was utilized, which included DNA extraction, PCR amplification and uninoculated culture controls. Negative controls remained negative throughout the study.

Inter-run variability was minimized by including amplification of a region of the HMBS reference housekeeping gene (as a sample DNA quality control), DNA extracted from a clinical case of *B. gibsoni* infection in a dog (as *Babesia* positive control DNA), naïve DNA from a human clinical case (as a negative control), and molecular-grade water (for reagent negative control) [8]. Cq values of HMBS gene amplification varied from mid 20-s to the high 30s when blood DNA and blood culture DNA were tested (resulting from a 1:10 dilution of blood to the enrichment blood culture media).

Amplified DNA products were purified and sequenced by Sanger’s method. Sequences were analyzed using Clustal W multi-sequence alignment (AlignX, Vector NTI Advanced 10.3.0 from Invitrogen) and compared to sequences previously deposited in the GenBank database using AlignX software (Vector NTI Suite 6.0, InforMax, Inc., Rockville, MD, USA). Samples with a qPCR Cq value lower than 40 were submitted for sequencing to confirm specific target amplification. Melt-curve assessment criteria included verification of a single, clean amplicon by visualizing a single, sharp peak in the derivative melt curve, and by confirming that the peak melting temperature was in the expected range for the product, i.e., 89.5–90.5 °C for *B. odocoilei*; 86.5 °C for *B. divergens*; 88.5 °C for *B. microti*; and 89 °C for *B. duncani*. As previously reported, Sanger sequencing (where primer sequences were excluded in the analysis) was performed, with continuous read length (between 476 bp and 567 bp depending on species detected) as well as chromatogram quality analysis to assess sequence quality and species identity [8]. 95% confidence intervals (95% CI) were calculated on the proportion of participants infected with *Babesia* spp., *Bartonella* spp., or both [13]. These were mathematically calculated using the following formula:

Standard Error (SE) = $\sqrt{\frac{p\left(1-p\right)}{n}}$, where *p* = the proportion of infected participants out of the total number of participants in that cohort (*n*). The margin of error (MOE) was calculated using a z-score of 1.96 multiplied by the SE. The 95% CI was then determined as the proportion +/− MOE.

## 3. Results

After retrospective questionnaire review, 50 individuals satisfied the entry criteria. There were 14 males and 36 females, ranging in age from 21 to 70 years, with a median age of 44 years. Of the 50 participants, 49 were white (one identified as Asian).

**Figure 2:**
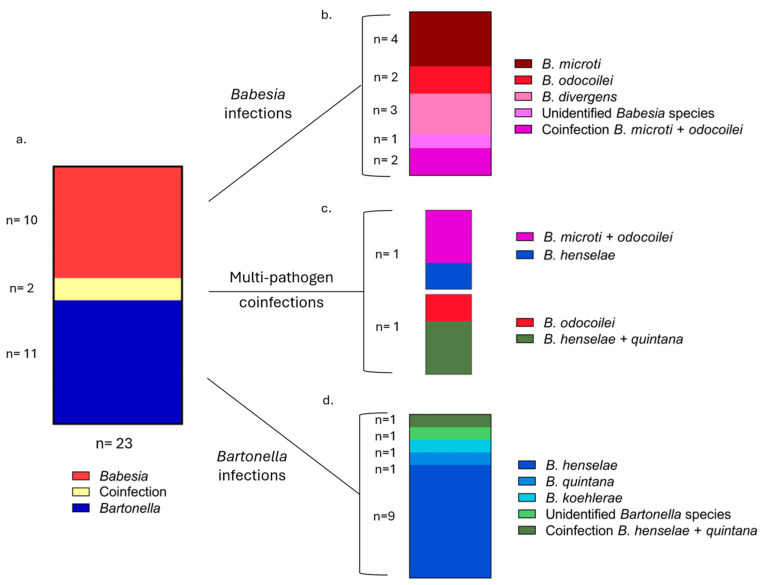
Overview of *Babesia* and *Bartonella* infections by species. Panel (**a**) depicts the number of individuals infected with only a *Babesia* sp., a *Bartonella* sp., or coinfected with a combination of both pathogens. Panel (**b**) indicates the number of participants infected with different *Babesia* spp., including coinfections with two *Babesia* spp. One individual coinfected with *Babesia microti* and *odocoilei* was also infected with *Bartonella henselae*, and one individual with a single *Babesia* species infection, *B. microti*, was coinfected with two species of *Bartonella*, *B. henselae* and *B. quintana* (**c**). Panel (**d**) displays the breakdown of individuals infected with various *Bartonella* species, including the two individuals infected with multi-pathogen coinfections indicated in panel (**c**). The number of individuals in each group (*n*) is indicated beside each panel.

Infection with *Babesia*, *Bartonella*, or *Babesia* and *Bartonella* co-infection was identified by DNA amplification in 23 of the 50 study participants (46%, 95% CI: 32–60%) (Figure 2).

*Babesia* and *Bartonella* DNA were not amplified from blood, serum or enrichment blood cultures from the remaining 27 (54%, 95% CI: 40–68%) individuals.

**Figure 3:**
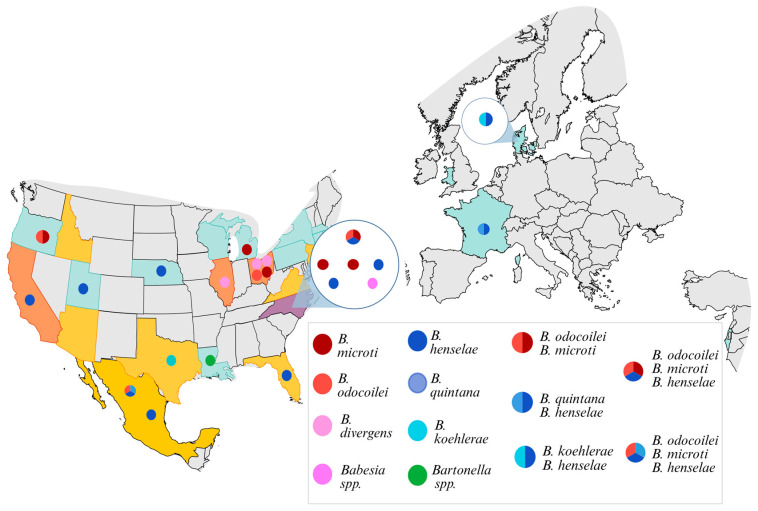
Map depicting the residency location and *Babesia* or *Bartonella* species detected in 23 study participants reporting chronic fatigue and neurological symptoms. Colors of states and/or countries signify the number of participants per area: purple = 9, orange = 4, yellow = 2, and blue = 1.

Study participants lived in six countries (Figure 3).

Demographics, illness duration and specialty physician visits for the cohort are summarized in Table 2. Infection with *Babesia* and *Bartonella* was documented most often in individuals from the southeastern and midwestern United States. Infection with *Babesia*, *Bartonella* or both genera was documented in individuals reporting fatigue from six months to longer than 10 years. Regardless of *Babesia* or *Bartonella* status (PCR+ or PCR−), nearly all individuals in the cohort had been evaluated by multiple medical specialists.

Study participants reported a broad spectrum of neurological symptoms. Difficulty remembering, headaches, insomnia, anxiety and irritability were the most frequently reported neurological symptoms among participants who were infected with either *Babesia*, *Bartonella*, or both species, or tested PCR-negative for *Babesia* and *Bartonella* DNA (Table 3).

Environmental exposures are summarized in Table 4. Nearly all individuals reported bites or scratches from animals, most often cats and dogs. Exposure to insects and arthropods was substantial regardless of PCR status. In the context of outdoor exposures, hiking was reported most often by individuals infected with *Babesia* species.

### Molecular Testing Results

Controls: *Babesia gibsoni* DNA, used as a positive *Babesia* amplification control, was not detected in any of the 586 blood or enrichment blood culture DNA extraction samples tested during the study. Similarly, *Babesia* and *Bartonella* DNA were not amplified from any DNA extraction or blood culture negative control sample processed concurrently with each participant’s blood and enrichment blood culture samples.

DNA Sequence/probe confirmation: Using Api18S rRNA-1690s and Api5.8S rRNA-20as (as forward and reverse primers), qPCR DNA amplification of the *Babesia* ITS1 region generated a single 450–650 bp band consistent with the expected DNA sizes (depending on species) for each of the three identified *Babesia* species. Similarly, using species-specific primers and probes, qPCR DNA amplification of the *Babesia* species-specific ITS1 region generated a single 150–225 bp band consistent with the expected DNA sizes (depending on species) for each of the three *Babesia* species (Table 5). Amplicon DNA sequence analysis of qPCR products or probe-based species-specific qPCR targeting the *Babesia* ITS1 region [8] confirmed infection with *Babesia microti* in four individuals (33%, 95% CI: 6–60%), *Babesia divergens*-like MO-1 in three individuals (25%, 95% CI: 0.5–49.5%), *Babesia odocoilei* in two individuals (17%, 95% CI: −4–38%), and co-infection with *B. microti* and *B. odocoilei* in two individuals (17%, 95% CI: −4–38%). The *Babesia* species (participant 40) could not be accurately determined due to a short, yet good-quality ITS1 DNA sequence that was a 70% match with *B. odocoilei*. (Table 5).

When targeting *Piroplasma* 18S rRNA (12 DNA extractions/participant), using the same primers and probes, 8 of the 50 individuals were positive by dPCR (16%, 95% CI: 6–26%) and 4 were positive by qPCR (8%, 95% CI: 0.5–15.5%). By targeting the *Piroplasma* ITS region, 18 of the 50 individuals were positive by qPCR (32%, 95% CI: 19–45%). The DNA sequence identity confirmed *Babesia* infection for 12 of the 18 individuals. For the six remaining ITS1-positive individuals, the sequences did not pass quality control for accurate species identification (even when the same samples were qPCR-positive during repeated testing). All eight of the 18S rRNA dPCR-positive individuals were ITS1 qPCR-positive, from which the infecting species was confirmed in five (63%, 95% CI: 30–96%) individuals by DNA sequencing.

Of the 13 individuals infected with a *Bartonella* sp., *B. henselae* DNA was amplified from blood or enrichment blood cultures from nine individuals, *B. quintana*, *B. koehlerae* and an undetermined *Bartonella* spp. in one individual each, and co-infection with *B. henselae* and *B. quintana* in one individual. DNA sequence similarities were generated by targeting the *Bartonella* 16S–23S intergenic spacer region (Table 6). Two participants were co-infected with a *Bartonella* and *Babesia* spp.; one participant was co-infected with *B. quintana*, *B. henselae* and *B. odocoilei*, while the other individual was co-infected with *B. henselae*, *B. odocoilei*, and *B. microti*.

For both genera, enrichment blood culture increased PCR detection sensitivity. *Babesia microti* and *B. divergens* detection occurred most often following enrichment blood culture, whereas *B. odocoilei* DNA was only amplified from blood (Table 7). Despite the use of species-specific primers, *B. duncani* DNA was not amplified from blood or enrichment blood cultures from any of the 50 individuals included in this study.

*Bartonella henselae* and *Bartonella quintana* DNA were variably amplified from blood or enrichment blood cultures incubated for 7, 14, and 21 days, while *B. koehlerae* and the uncharacterized *Bartonella* DNA were only amplified from blood (Table 8).

## 4. Discussion

### 4.1. Symptomatology and Pathogen Framework

The rationale for this cohort study was to determine the frequency of infection with *Babesia* spp., *Bartonella* spp. or both genera in sick individuals reporting fatigue for at least six months’ duration. The duration of illness in this cohort ranged from 6 months to longer than 10 years. Fatigue is a common symptom, which is associated with many illnesses, both infectious and noninfectious in etiology. When fatigue becomes chronic in nature and the disease etiology cannot be determined, diagnostic evaluations become extensive, medical management becomes challenging, and patients suffer from an inability to perform daily functions. Despite decades of research, an infectious cause of ME/CFS has not been definitively determined [10,11]. The authors are not aware of studies that address a potential role for chronic bloodborne bacteria (*Bartonella* spp.) or protozoa (*Babesia* spp.) species as a cause or cofactor in fatigue, consistent in duration with ME/CFS. It is likely that ME/CFS has numerous etiologies, perhaps including polymicrobial infections consisting of bacteria, protozoa, viruses and other pathogenic organisms.

Among participants, 23/50 (46%, 95% CI: 32–60%) individuals were infected. Using targeted qPCR amplification and DNA sequencing, infection with *Babesia* spp., *Bartonella* spp. or both genera was confirmed in 10, 11, and 2 individuals reporting chronic fatigue, respectively. This small cohort study suggests that persistent infection or co-infections with these genera may represent a cause of sustained fatigue and neurological abnormalities. As previously reported, anxiety and irritability were reported more often in this study by individuals infected with a *Bartonella* species [14].

Human babesiosis, a disease caused by a parasitic intraerythrocytic infection of red blood cells, is an emerging, zoonotic, tickborne disease of increased prevalence in the United States and throughout much of the world [15,16,17,18,19]. The infection is caused by members of the genus *Babesia* of the order *Piroplasmida* (Apicomplexa phylum), a genus comprising over 100 species, of which at least 8 species or sub-species have infected people on the basis of DNA sequence evidence [8]. Acute human babesiosis is predominantly characterized by fatigue, fever, sweating, chills, generalized myalgia, sleep disorders, and hepatosplenomegaly. Severe symptoms such as hemolytic anemia and neurological complications (including headache, syncope, neuropathy, confusion, vertigo, and coma) have been reported, most frequently in patients with comorbidities such as immunodeficiencies (splenectomy, infection with HIV/AIDS, or patients receiving immunosuppressive drugs), diabetes, cancer, chronic heart, lung, kidney, or liver diseases [20,21,22,23]. The tick-transmitted *Babesia* species documented by DNA sequencing in this study occurred in individuals from the United States and Mexico, known geographic regions for human infection with these organisms.

Neurobartonelloses represent a broad spectrum of neurological diseases caused by infection with a *Bartonella* species [24]. Historically, various neurological presentations have most often been reported as atypical manifestations of Cat Scratch Disease (CSD), caused by *B. henselae.* Fatigue and chronic neurological symptoms have been reported in people who lack a history of fever, lymphadenopathy, and cat contact (CSD) [25,26,27]. Seizures, encephalitis, transverse myelitis, peripheral neuropathy (Guillain–Barré syndrome), psychoses and schizophrenia have been associated with *Bartonella* infections. Like infection with *Babesia* species, healthy individuals and blood donors can be occultly infected, which complicates the medical assessment of the role or importance of these infections when confirmed in individual patients. Also, like babesiosis, bartonellosis, first recognized in North America among immunocompromised HIV-infected individuals with bacillary angiomatosis, has been diagnosed in patients in association with splenectomy, organ transplantation recipients and patients receiving immunosuppressive drugs [28]. Immunosuppression likely decreases the suppression of these intracellular organisms, resulting in enhanced PCR detection in blood. Based upon survey questionnaire responses, no individual infected with *Babesia*, *Bartonella* or both genera in this study reported infection with AIDS, having had a splenectomy, or a concurrent diagnosis of diabetes or active cancer. The *Bartonella* species sequenced from participants in this study are transmitted by several arthropod vectors [28]. Specifically, *B. henselae* and *B. koehlerae* are transmitted by fleas (*Ctenocephalides felis*), which have a worldwide distribution. Infection with *B. quintana*, transmitted primarily by the human body louse, has been reported from Central America, France and North America [28].

### 4.2. Diagnostic Considerations in Pathogen Detection

As previously reported, limitations in diagnostic sensitivity for the detection of *Babesia* and *Bartonella* DNA are a major consideration when assessing blood infection and enrichment blood culture testing [1,12,29]. While *B. odocoilei* was detected only following blood DNA extraction, infection with *B. divergens*-like MO-1 and *B. microti* were most often confirmed after blood enrichment culture for 7 to 21 days (Table 7). Presumably, these two *Babesia* species remained viable and multiplied during liquid enrichment culture incubation. Based upon a 10-fold dilution of blood into liquid culture medium, the original blood DNA extraction would have been 10× more concentrated when compared with DNA extractions from the liquid cultures. Although amplification of trace DNA in cultures cannot be ruled out, recent publications reporting in vitro culture of *Babesia* organisms support the possibility of intraerythrocytic proliferation, an area requiring additional study [30,31,32]. Based upon the blinded case selection and predetermined study design, 4 of the 12 *Babesia*-infected individuals in this study had been included from two prior publications [1,3]. Repeat blinded testing redocumented infection with *B. odocoilei* in two of three previously diagnosed individuals and *B. divergens*-like MO-1 in one individual. Co-infection with *B. microti*, not amplified from two previously reported *B. odocoilei*-infected individuals, provided additional evidence for co-infection with more than one *Babesia* species and further illustrates challenges with molecular detection of co-infections with these protozoal organisms [3].

Similarly, while *B. koehlerae* was detected in the blood of a single individual, most *Bartonella* infection was confirmed after enrichment blood culture for 7 to 21 days, as previously reported [33].

DNA amplification, using various molecular methods, has become the preferred methodology for the diagnosis of babesiosis and bartonellosis. In contrast to serology, PCR provides direct evidence of infection [3,8]. Previous studies have documented increased sensitivity for the diagnosis of bartonellosis when enrichment blood culture was combined with qPCR or dPCR testing and when three, rather than one, blood specimens were obtained within a 7-day period [1,12,33,34]. Three blood collections within a 7-day period were provided by individuals entering this research study. DNA extracted from blood, serum, and three 7-day, 14-day and 21-day enrichment blood cultures generated fifteen samples for qPCR and dPCR testing per study participant. Rarely were more than three or four of the fifteen DNA extractions from a patient PCR-positive. Due to enhanced sensitivity, dPCR positivity consistently exceeded qPCR positivity. The low parasitemia and bacteremia associated with these two genera impairs diagnostic sensitivity, thereby hindering acquisition of an accurate diagnosis. Among many others, two case reports illustrate the diagnostic complexity associated with these chronic intravascular infections. Infection with *B. henselae*, *B. odocoilei*, and *B. divergens*-like/MO-1 was documented in an 8-year-old boy from Canada by PCR amplification and sequencing of DNA extracted from blood and enrichment brain biopsy cultures [1]. Based upon molecular testing results, five family members (father, mother, two daughters and a son) and a pet dog were infected with *B. divergens*-like MO-1, both parents were infected with *B. microti*, and all family members, both pet dogs, and fleas from one pet rabbit were infected with *Bartonella* (*B. quintana* and/or *B. henselae*, or an undetermined *Bartonella* species) [2]. Although chronically ill, this cohort was heterogenous in the context of age, sex, geographic origin, symptomatology, overall duration of illness, prior medical evaluations, and therapeutic interventions. Testing in this study was limited to two genera. As *Babesia* species are transmitted to humans by ticks belonging to the *Ixodes* genus, future prospective studies of chronic fatigue syndrome patients should also consider infection with the *Borrelia* Lyme Group (generally not spirochetemic) and *Borrelia miyamotoi*, a spirochetemic member of the Hard Tick-Borne Relapsing Fever Group. Documentation of *Babesia*, *Bartonella* or DNA of both genera does not confirm causation for the fatigue or neurological symptoms that were self-reported. As the timing and mode of infection could not be determined, the duration of infection compared to the duration of illness is unknown.

In conclusion, despite its retrospective nature, this study documented infections with *Babesia*, *Bartonella* or both organisms in nearly half of a cohort reporting chronic fatigue and neurological abnormalities. Future case–control, multicenter studies are needed to determine whether *Babesia* and *Bartonella* species cause or are cofactors in patients diagnosed with ME/CFS. Ongoing improvements in molecular diagnostic documentation of individual or coinfections with these piroplasm and bacterial organisms will facilitate directed antimicrobial treatment, thereby potentially improving patient outcomes.

## Figures and Tables

**Table 1:** Primers and probes used for the amplification and identification of *B. divergens*, *B. duncani*, *B. microti*, and *B. odocoilei* 18SrRNA-5.8SrRNA intergenic spacer region in this study.

*Babesia* ITS1 Target	Primers/Probe Designation	DNA Sequences
*Babesia* genus ITS1 region (550–650 bp)	Api18S rRNA-1690s	5′ CTCCTACCGATCGAGTGATCCGGT 3′
	Api5.8S rRNA-20as	5′ GCTGCGTCCTTCATCGTTGTGTGAG 3′
*B. divergens* ITS1 region (225 bp)	Bdivergens ITS1-25s	5′ CTCGGCTTCGACATTTACGTTGTGTAAGCT 3′
	Bdivergens ITS1-150as	5′ CAACTACAGTAGTTACACCGYAGTAARCATAC 3′
	Probe Bdivergens ITS1-70	5′ HEX CTTTTKGTGGTTTCGTATTTGYCGTTG BHQ2 3′
*B. duncani* ITS1 region (170 bp)	Bduncani ITS1-1s	5′ GTGTTTAAACCGCGCTTATGCGCAGGTC 3′
	Bduncani ITS1-130as	5′ CTGCACTGGCGGGGTGAAAAGTAAC 3′
	Probe Bduncani ITS1-80	5′ Cy5-TGGCTTTGCGGTTCGCCGTACGGCCCC-BHQ3 3′
*B. microti* TS1 region (185 bp)	Bmicroti ITS1-25s	5′ TATCAGAGTTCTTTGTATCCCATTTGGGTTA 3′
	Bmicroti ITS1-160as	5′ GAAAATACCTTGGGAGTGAGAACGCCCCGT 3′
	Probe Bmicroti ITS1-70	5′ CalFluoRed590-AGAAGAGTGGCCTTGGACGTAG-BHQ2 3′
*B. odocoilei* ITS1 region (150 bp)	Bodoco ITS1-100s	5′ CTGTTGCACTTTTGTGCTTGACGTTGT 3′
	Bodoco ITS1-255as	5′ CAAGCGCAGGGATGGAAACGGA 3′
	Probe Bodoco ITS1-200	5′ FAM-GGCCTCGTCATGGCGACGTGGT-BHQ1 3′

bp = expected base pair amplicon size.

**Table 2:** Demographic, illness duration, and physician information reported by participants (*n* = 50) who were infected with either *Babesia*, *Bartonella*, *Babesia* and *Bartonella*, or tested PCR-negative for *Babesia* and *Bartonella* DNA.

Characteristic			Overall Study Population No. (%)	*Babesia* ONLY PCR Positive No. (%)	*Bartonella* PCR ONLY Positive No. (%)	Co-Infection No. (%)	Negative PCR No. (%)
Total			50 (100)	10 (20)	11 (22)	2 (4)	27 (54)
Sex	F		36 (72)	8 (80)	7 (64)	2 (100)	19 (70)
	M		14 (28)	2 (20)	4 (36)	0 (0)	8 (30)
Region of Residence	NE USA		7 (14)	0 (0)	0 (0)	0 (0)	7 (26)
	SE USA		15 (30)	3 (30)	4 (36)	1 (50)	7 (26)
	MW USA		11 (22)	6 (60)	1 (9)	0 (0)	4 (15)
	SW USA		3 (6)	0 (0)	1 (9)	0 (0)	2 (7)
	W USA		8 (16)	1 (10)	2 (18)	0 (0)	5 (19)
	Other countries		6 (12)	0 (0)	3 (27)	1 (50)	2 (7)
Duration of Illness	≥6 months		20 (40)	4 (40)	5 (45)	0 (0)	11 (41)
	≥5 Years		13 (26)	2 (20)	3 (27)	1 (50)	7 (26)
	≥10 Years		17 (34)	4 (40)	3 (27)	1 (50)	9 (33)
Specialty Physician Visits	Y		42 (84)	5 (50)	10 (91)	2 (100)	25 (93)
	N		8 (16)	5 (50)	1 (9)	0 (0)	2 (7)
	Type	ID	22 (44)	2 (20)	5 (45)	0 (0)	15 (56)
		GI	25 (50)	3 (30)	7 (64)	0 (0)	15 (56)
		RH	22 (44)	1 (10)	6 (54)	0 (0)	15 (56)
		NE	33 (66)	4 (40)	9 (82)	2 (100)	18 (67)
		HE	6 (12)	1 (10)	1 (9)	0 (0)	4 (15)

ID = infectious disease; GI = gastroenterologist; RH = rheumatologist; NE = neurologist; HE = hematologist.

**Table 3:** The five most frequent neurological symptoms reported by participants (*n* = 50) who were infected with either *Babesia*, *Bartonella*, *Babesia* and *Bartonella*, or tested PCR-negative for *Babesia* and *Bartonella* DNA.

Total DNA Positive and Negative Participants	Difficulty Remembering	Headache	Insomnia	Anxiety	Irritability
*Babesia* (*n* = 10)	8	5	6	3	3
*Bartonella* (*n* = 11)	10	10	8	9	9
*Babesia* and *Bartonella (n* = 2)	2	2	1	1	1
Test Negative (*n* = 27)	17	19	18	14	15

**Table 4:** Environmental exposure information reported by participants (*n* = 50) who were infected with either *Babesia*, *Bartonella*, or both, or tested PCR-negative for *Babesia* and *Bartonella* DNA.

Characteristic			Overall Study Population No. (%)	*Babesia* ONLY PCR Positive No. (%)	*Bartonella* PCR ONLY Positive No. (%)	Co-Infection No. (%)	Negative PCR No. (%)
Animal Contact	Y		47 (94)	10 (100)	11 (100)	2 (100)	24 (89)
	N		3 (6)	0 (0)	0 (0)	0 (0)	3 (11)
Animal bites/scratches	Y		39 (78)	9 (90)	8 (73)	2 (100)	20 (74)
	N		11 (22)	1 (10)	3 (27)	0 (0)	7 (26)
	Type	Dog	31 (62)	8 (80)	5 (45)	2 (100)	16 (59)
		Cat	34 (68)	8 (80)	7 (64)	2 (100)	17 (63)
		Bird	12 (24)	4 (40)	2 (18)	2 (100)	4 (15)
		Horse	6 (12)	2 (20)	0 (0)	1 (50)	3 (11)
		Rodent	17 (34)	5 (50)	2 (18)	1 (50)	9 (33)
Insect exposure	Mosquitos		47 (94)	10 (100)	10 (91)	2 (100)	26 (96)
	Tick		41 (82)	9 (90)	9 (82)	1 (50)	22 (81)
	Flea		39 (78)	10 (100)	7 (64)	2 (100)	20 (74)
	Biting Fly		32 (64)	7 (70)	7 (64)	1 (50)	17 (63)
	Lice		23 (46)	4 (40)	4 (36)	1 (50)	14 (52)
	Spiders		36 (72)	8 (80)	7 (64)	2 (100)	19 (70)
	Scabies		6 (12)	1 (10)	1 (9)	0 (0)	5 (19)
	Bedbugs		6 (12)	0 (0)	2 (18)	0 (0)	4 (15)
Outdoor exposure	Hiking		38 (76)	9 (90)	8 (73)	1 (50)	20 (74)
	Wildlife Rescue		6 (12)	3 (30)	2 (18)	1 (50)	0 (0)
	Hunting		5 (10)	1 (10)	0 (0)	1 (50)	3 (11)
	Other		39 (78)	9 (90)	8 (73)	1 (50)	21 (78)

**Table 5:** *Babesia* species and respective sequence homologies for 12 participants reporting fatigue of at least six months’ duration and neurological symptoms. Note: ^ species-specific probe signal detected but a sequence was not obtained; * partially readable *Babesia* sequence, but inadequate for species determination.

Participant	Species	Homology and Reference Sequence
4	*B. odocoilei*	376/385 bp (97.7%) *B. odocoilei* AY339756
5	*B. odocoilei*	101/105 bp (96.2%) *B. odocoilei* AY339756
	*B. microti*	103/103 bp (100%) *B. microti* GU230755
8	*B. microti*	520/524 bp (99.2%) *B. microti* GU230755
11	*B. microti*	453/455 bp (99.6%) *B. microti* GU230755
19	*B. divergens* MO-1	475/477 bp (99.6%) *B. divergens* PQ184854
22	*B. odocoilei*	123/124 bp (99.2%) *B. odocoilei* AY158711
24	*B. microti*	473/476 bp (99.4%) *B. microti* GU230755
25	*B. divergens* MO-1	564/567 bp (99.5%) *B. divergens* PQ184854
27	*B. divergens* MO-1	564/567 bp (99.5%) *B. divergens* PQ184854
30	*B. odocoilei*	Probe based ^
	*B. microti*	Probe based ^
40	*Babesia* sp. *	50/50 bp * (100%) *B. odocoilei* AY339756
41	*B. microti*	119/121 bp (98.4%) *B. microti* GU230755

**Table 6:** *Bartonella* species and respective sequence homologies for 13 participants reporting fatigue of at least six months duration and neurological symptoms. Note: ^ species-specific probe signal detected but a sequence was not obtained; * partially readable *Bartonella* sequence, but inadequate for species determination.

Participant	Species	Homology and Reference Sequence
1	*B. henselae*	126/127 bp (99.2%) *B. henselae* SA2 AF369529
4	*B. henselae*	138/138 bp (100%) *B. henselae* SA2 AF369529
	*B. quintana*	128/128 (100%) *B. quintana* Oklahoma AF368391
5	*B. henselae*	140/140 bp (100%) *B. henselae* SA2 AF369529
6	*B. henselae*	135/135 bp (100%) *B. henselae* SA2 AF369529
14	*B. quintana*	129/129 bp (100%) *B quintana* Oklahoma AF368391
15	*B. henselae*	Probe based ^
17	*B. henselae*	128/128 bp (100%) *B. henselae* SA2 AF369529
18	*B. koehlerae*	110/110 bp (100%) *B. koehlerae* AF312490
31	*B. henselae*	122/123 bp (99.2%) *B. henselae* SA2 AF369529
43	*B. henselae*	Probe-based ^
44	*B. henselae*	Probe-based ^
45	*B. henselae*	135/135 bp (100%) *B. henselae* SA2 AF369529
46	*B.* spp. *	87/87 (100%) *Bartonella* spp.

**Table 7:** Number of *Babesia*-positive individuals per DNA sample source. Note: for some individuals *Babesia* DNA was detected in more than one sample type from the same individual (i.e., DNA amplified from blood and from one or more blood enrichment cultures at 7, 14, or 21 days).

	Sample Source			
	Blood	Blood Cult 7 Days	Blood Cult 14 Days	Blood Cult 21 Days
*B. odocoilei*	4	-	-	-
*B. microti*	1	5	-	1
*B. divergens*	1	-	2	1
*Babesia* spp.	-	-	1	-

**Table 8:** Number of *Bartonella*-positive individuals per DNA sample source. Note: for some individuals *Bartonella* DNA was detected in more than one sample type from the same individual (i.e., DNA amplified from blood and from one or more blood enrichment cultures at 7, 14, or 21 days).

	Sample Source			
	Blood	Blood Cult 7 Days	Blood Cult 14 Days	Blood Cult 21 Days
*B. henselae*	8	2	2	4
*B. quintana*	-	1	1	1
*B. koehlerae*	1	-	-	-
*Bartonella new* spp.	1	-	-	-

## Data Availability

The data presented in this study is available on request from the corresponding author. This data is restricted due to human health data privacy concerns.
